# Dietary supplementation of propionylated starch to domestic cats provides propionic acid as gluconeogenic substrate potentially sparing the amino acid valine

**DOI:** 10.1017/jns.2014.18

**Published:** 2014-08-13

**Authors:** Kristel Rochus, An Cools, Geert P. J. Janssens, Lynn Vanhaecke, Birgitte Wuyts, Trevor Lockett, Julie M. Clarke, Veerle Fievez, Myriam Hesta

**Affiliations:** 1 Laboratory of Animal Nutrition, Department of Nutrition, Genetics and Ethology, Faculty of Veterinary Medicine, Ghent University, Heidestraat 19, B-9820 Merelbeke, Belgium; 2 Laboratory of Chemical Analysis, Department of Veterinary Public Health and Food Safety, Faculty of Veterinary Medicine, Ghent University, Salisburylaan 133, B-9820 Merelbeke, Belgium; 3 Laboratory of Metabolic Disorders, University Hospital Ghent, De Pintelaan 185, B-9000 Ghent, Belgium; 4 Preventive Health National Research Flagship and CSIRO Animal, Food and Health Sciences, PO Box 52, North Ryde, NSW 1670, Australia; 5 Department of Animal Production, Faculty of Bioscience Engineering, Ghent University, Proefhoevestraat 10, B-9090 Melle, Belgium

**Keywords:** Acylated starch, Domestic cats, Fermentation, Gluconeogenesis, Propionic acid, DS, degree of substitution, HAMSA, acetylated high-amylose maize starch, HAMSP, propionylated high-amylose maize starch

## Abstract

In strict carnivorous domestic cats, a metabolic competition arises between the need to use amino acids for gluconeogenesis and for protein synthesis both in health and disease. The present study investigated the amino acid-sparing potential of propionic acid in cats using dietary propionylated starch (HAMSP) supplementation. A total of thirty cats were fed a homemade diet, supplemented with either HAMSP, acetylated starch (HAMSA) or celite (Control) for three adaptation weeks. Propionylated starch was hypothesised to provide propionic acid as an alternative gluconeogenic substrate to amino acids, whereas acetic acid from HAMSA would not provide any gluconeogenic benefit. Post-adaptation, a 5-d total faecal collection was carried out to calculate apparent protein digestibility coefficients. Fresh faecal and blood samples were collected to analyse fermentation endproducts and metabolites. The apparent protein digestibility coefficients did not differ between supplements (*P* = 0·372) and were not affected by the protein intake level (*P* = 0·808). Faecal propionic acid concentrations were higher in HAMSP than in HAMSA (*P* = 0·018) and Control (*P* = 0·003) groups, whereas concentrations of ammonia (*P* = 0·007) were higher in HAMSA than in HAMSP cats. Tendencies for or higher propionylcarnitine concentrations were observed in HAMSP compared with HAMSA (*P* = 0·090) and Control (*P* = 0·037) groups, and for tiglyl- + 3-methylcrotonylcarnitine concentrations in HAMSP as compared with Control (*P* = 0·028) cats. Methylmalonylcarnitine concentrations did not differ between groups (*P* = 0·740), but were negatively correlated with the protein intake level (*r* –0·459, *P* = 0·016). These results suggest that HAMSP cats showed more saccharolytic fermentation patterns than those supplemented with HAMSA, as well as signs of sparing of valine in cats with a sufficient protein intake.

Intestinal microbial fermentation and the consequent production of metabolites, such as SCFA, are considered to be beneficial for most animals^(^^1^^)^, even in a strict carnivorous species like the domestic cat^(^^2^^)^. In particular, the amino acid-sparing potential of fermentation-derived propionic acid, hypothesised by Verbrugghe *et al.*^(^^3^^–^^5^^)^, may be advantageous for cats in both health and disease conditions. Propionic acid, produced upon intestinal microbial fermentation of carbohydrates, can be used as an alternative gluconeogenic substrate in domestic cats^(^^6^^)^, potentially reducing the demand on amino acids for gluconeogenesis. Guar gum fermentation has been shown to produce high concentrations of propionic acid upon incubation with faecal inoculum from cats fed two diets, contrasting in both protein and fibre concentrations and sources^(^^7^^)^. The high viscosity or the small-intestinal fermentation of this soluble fibre supplement, however, appeared to have impaired the assessment of the amino acid-sparing potential of propionic acid^(^^8^^)^. Therefore, another approach for supplying the liver with fermentation-derived propionic acid was searched for.

Acylated starches are comprised of either low- or high-amylose maize starch that has been esterified with acetic, propionic or butyric acid to a high degree of substitution (DS) (DS between 0·2 and 0·3)^(^^9^^)^, where DS is defined as the number of hydroxyl groups on each D-glucopyranosyl unit derivatised by substituent groups^(^^10^^,^^11^^)^. These modified starches are classified as resistant starch type 4, as they are only partially digestible in the small intestine of rats^(^^12^^–^^14^^)^ and humans^(^^10^^)^. In the large intestine, however, the ester bond can be cleaved by bacterial enzymes, releasing the coupled SCFA. The residual starch carrier is then available for fermentation by the intestinal microbiota as well, leading to further production of SCFA^(^^9^^,^^10^^)^. While acylated starches have never been used in feline nutrition or research before, their potential to provide significant concentrations of a specific desirable SCFA to the large intestine has been shown in both rodents and human subjects^(^^9^^–^^15^^)^. The aims of the present study were to examine the potential of dietary propionylated starch to enhance the delivery of propionic acid to the feline large intestine, and to assess the consequent amino acid-sparing capabilities of the absorbed propionic acid in domestic cats in a model applicable to clinical and practical circumstances (dietary supplementation of a fibre source).

## Materials and methods

### Animals

A total of thirty healthy adult domestic shorthair cats (fifteen female and fifteen male), with a mean body weight and age of 4·0 (sd = 0·9) kg and 5·6 (sd = 3·0) years, respectively, were included in the present study. All cats were castrated, except for five females that remained intact. Before inclusion in the study, the cats were declared healthy based on a thorough physical examination and complete blood count and serum biochemistry analyses. The cats were divided into three groups (two treatment groups and one control group), consisting of ten cats each (*n* 10), considering equal distribution of age, body weight, body condition score^(^^16^^)^, BMI^(^^17^^)^ and neuter state.

### Experimental design and diet

All cats were fed on the same homemade diet (see below) to fulfil maintenance energy requirements (418·4 kJ/kg^0·67^; National Research Council^(^^18^^)^) during a 3-week adaptation period with two isoenergetic meals per d in individual housing. They were weighed weekly to enable adjustments of the food amounts until amounts needed to maintain stable body weight were achieved. At all times, cats had *ad libitum* access to tap drinking water provided by automatic drinking fountains and refreshed daily. The cats were group-housed between meals with a maximum of ten cats per group (randomised for housing, not housed per treatment group). The experimental protocol was approved by the Ethical Committee of the Faculty of Veterinary Medicine, Ghent University, Belgium (EC 2012/06), and was in accordance with institutional and national guidelines for the care and use of laboratory animals.

A homemade diet was formulated, and the ingredient composition and the analysed nutrient content are depicted in Table 1. The homemade diet consisted of cooked (boiled in water for 10 min) chicken breast meat (without skin, bones and visible fat) as the protein source and white rice (steam cooked separately for 10 min) as the main carbohydrate source. After cooking, the chicken and rice were thoroughly ground and mixed. Then, the mixture was divided into approximate daily portions pooled for all cats, and frozen at –20°C. Every day one pooled portion was transferred to 4°C to be defrosted gradually over a 2-d period. The day before feeding, the pooled portion was accurately weighed and subdivided into individual portions. In total, diets were kept 3 d at 4°C until being fed to the animals. Before feeding, the portions were allowed to warm to room temperature and blended with rapeseed oil (8·1 % of total food amount (TFA); Vandemoortele koolzaadolie; Vandemoortele Lipids nv.), custom-formulated vitamin–mineral premix (1·0 % TFA; Laboratory of Animal Nutrition and Dietetics, Veterinary Sciences Department, University of Munich), and the experimental or control supplement, each at 4 % of dietary DM. The experimental supplements were propionylated (DS 0·24) or acetylated (DS 0·23) Hylon VII high-amylose maize starch (HAMSP and HAMSA, respectively), both prepared by the National Starch & Chemical Company. Since in general acetic acid is not a gluconeogenic substrate^(^^19^^)^, the HAMSA supplement was used as a negative control for gluconeogenic amino acid sparing, when compared with HAMSP, which is hypothesised to provide the liver with additional gluconeogenic propionic acid as compared with the baseline propionic acid yield from fermentation of the residual starch carrier in both HAMSP and HAMSA. The control supplement was celite (Celite 545; VWR International), which consists of non-digestible and non-fermentable ash^(^^20^^)^.

**Table 1.** Ingredients and macronutrient composition analysis of the experimental diets fed to thirty domestic shorthair cats to study the amino acid-sparing potential of propionic acid

Ingredients/nutrients	HAMSP*	HAMSA†	Control‡
Ingredients (% of total food amount)			
Chicken breast meat	55·0	55·0	55·0
White rice§	34·9	34·9	34·9
Rapeseed oil‖	8·1	8·1	8·1
Vitamin–mineral supplement¶	1·0	1·0	1·0
Experimental/control supplement	1·4	1·4	1·4
Nutrients (analysed)			
DM (% as-is)	39·4	39·4	39·4
Crude protein (% DM)	24·6	24·9	24·2
Crude fat (% DM)	18·9	18·6	17·0
Crude ash (% DM)	2·5	2·6	5·9
Crude fibre (% DM)	0·3	0·2	0·2
NFE (% DM)**	53·7	53·7	52·7
Total dietary fibre (% DM)	2·8	3·4	1·9
ME (kJ/100 g as fed)††	747·3	746·0	713·8

HAMSP, propionylated high-amylose maize starch; HAMSA, acetylated high-amylose maize starch; NFE, N-free extract; ME, metabolisable energy.

* HAMSP: Propionylated Hylon VII high-amylose maize starch (National Starch & Chemical Company).

† HAMSA: Acetylated Hylon VII high-amylose maize starch (National Starch & Chemical Company).

‡ Control: Celite 545 (VWR International).

§ White rice: dessert rice, Horeca Select (Makro).

‖ Rapeseed oil: Vandemoortele koolzaadolie (Vandemoortele Lipids nv.).

¶ Calculated and produced by the Laboratory of Animal Nutrition and Dietetics, Veterinary Sciences Department, University of Munich. Composition (% as-is): Ca, 12·0; P, 1·3; Na, 0·5; K, 11·1; Mg, 0·7; Cl, 1·0; taurine, 15; Cu, 0·02; I, 0·0058; Fe, 0·28; Mn, 0·02; Zn, 0·28.

** NFE (% DM) was calculated as 100 – crude protein – crude fat – crude ash – crude fibre, with all components on a DM basis.

†† Estimated using a four-step calculation^(^^19^^)^.

### Sampling

After an overnight fast (12 h), preprandial blood samples were aseptically drawn from the jugular vein before the study (general blood work for inclusion of the cats in the study, see above) and on the first day of the collection period (week 4). Immediately after collection, blood samples were placed into Vacutainer^®^ tubes containing lithium heparin or serum clot activator, and serum and plasma were separated by centrifugation (10 min at 1620 ***g***) and frozen at –20°C until analyses.

After the blood sampling, the cats were housed individually and for 5 d all faeces were collected. During the adaptation period, the cats were housed individually during meals and trained during this period of time to use the litter box with gradually decreasing amounts of litter inside. For faecal sample collection, no litter was present in the litter boxes. Faecal samples were collected from the litter boxes five times daily and frozen at –20°C. During the total collection period, the cats were also monitored for fresh faecal samples, and at least one fresh faecal sample per cat was collected within 30 min of voiding. Immediately before freezing (–20°C), the fresh faecal samples were scored according to the Purina Faecal Scoring System for dogs and cats^(^^21^^)^, and the faecal pH was measured as described in Rochus *et al.*^(^^8^^)^.

### Chemical analyses

The experimental diets were subjected to proximate and total dietary fibre analyses as in Rochus *et al.*^(^^8^^)^, and the analyses of faecal SCFA and ammonia (NH_3_) were performed as previously described as well^(^^8^^)^. The phenolic compounds, indole, *p*-cresol, and phenol, were extracted from fresh faecal samples by mixing 0·25 g of faeces (defrosted overnight at 4°C) with 40 µl of internal standard (100 ng/μl 5-methylindole) and 1960 µl of methanol. The mixture was vortexed for 30 s, ultrasonically vibrated, rotated^(^^4^^)^, then centrifuged for 10 min at 13 300 ***g***. Supernatant fractions were collected and reduced to a volume of 200 µl by evaporation. After another 10 min centrifugation (13 300 ***g***), a subsample of 60 µl was combined with 140 µl of water. A quantity of 10 µl of this dilution was injected on a liquid chromatography system consisting of a Thermo Fisher Scientific Accela U-HPLC pumping device, coupled with an Accela Autosampler and Degasser. Chromatographic separation was achieved on an HSS-C18 column (1·8 µm; 50 mm × 2·1 mm) (Waters), kept at 40°C. The mobile phase, consisting of 50 mm-ammonium acetate and acetonitrile, was pumped isocratic (i.e. the same proportions of solvents are used throughout the entire run instead of using a gradient) at a flow rate of 0·3 ml/min for 10 min. Detection was performed on a photodiode array (PDA) detector (Thermo Fisher Scientific) at 270 nm. Remaining faecal samples of total collections were lyophilised and pooled per cat per period. Pooled faeces were sieved through a 1 mm mesh for hair removal, ground up in a grinding mill (1 mm mesh, Brabender Rotary Mill; Brabender GmbH and Company KG), and proximate analyses as well as analyses of bacterial N were performed as previously described^(^^8^^)^. Plasma acylcarnitine and amino acid profiles, 1- and 3-methylhistidine were analysed as previously described^(^^8^^)^. Serum urea, creatinine, total protein and plasma creatine kinase were analysed spectrophotometrically (ARCHITECT c Systems and the AEROSET System; Abbott Products sa/nv) using commercial kits (Urea N, Creatinine, Total protein, and Creatine kinase kit; Abbott Products sa/nv). The DS of the acylated starches was determined by using ^13^C-NMR spectroscopy (DRX-500 spectrometer; Bruker), by using the resolution of the six glucose carbons as assigned by Dais & Perlin^(^^22^^)^.

### Calculations

Apparent protein digestibility coefficients were calculated based on dietary nutrient intake and faecal nutrient excretion based on total faecal collection^(^^8^^)^. Energy, crude protein, crude fat and amino acid intake were calculated per cat per kg metabolic weight per d. In the plasma acylcarnitine profile, the ratio of the concentrations of methylmalonyl- and propionylcarnitine was calculated.

### Statistical analysis

For all statistical analyses, SPSS version 21 (IBM) was used. Statistical significance was set at *P* < 0·05. Before further analyses of all data, normality was examined using the Kolmogorov–Smirnov test on standardised residuals (*P* > 0·01). Homogeneity of variances was tested by means of the Levene's test for equality of error variances. If the significance of the latter test was below 0·05, a logarithmic transformation of the data was done, which resolved the variance heterogeneity in most cases (faecal propionic acid and phenol excretions and concentrations, methylmalonylcarnitine:propionylcarnitine ratio, plasma creatine kinase). If this transformation did not restore the homogeneity of variance, the data were analysed non-parametrically (see below; apparent protein digestibility, 3-hydroxy (OH) 3-methylglutarylcarnitine, 3-OH isovaleryl- + 2-methyl-3-OH butyrylcarnitine). Outliers in the normally distributed data were detected if the standardised values (*Z* scores) exceeded a value of ((*n* – 1)/√*n*)^(^^23^^)^. All normally distributed data were analysed using a univariate ANOVA to test the effects of supplement (HAMSP, HAMSA, Control) with the protein intake level as a covariate. This covariate was included in the model to correct for the fact that more than half of the cats had a protein intake below the recommended minimal requirements (3·97 g/kg^0·67^; see National Research Council^(^^18^^)^), which might bias the results. The differences between treatments were unravelled using Fisher's least significant difference (LSD) *post hoc* test. If the covariate protein intake level was significant for a specific parameter, Pearson product moment correlation coefficients were calculated between the protein intake level and the respective parameter. Data that were not normally distributed (faecal pH) were analysed non-parametrically by means of Kruskal–Wallis tests for independent samples, again with supplement as a factor. As far as protein intake level, non-normally distributed data were divided into two categories: protein intake below (category 1 = PIB) and above (category 2 = PIA) the recommended minimal requirement (3·97 g/kg^0·67^/d; see National Research Council^(^^18^^)^). This grouping was done irrespective of treatment and resulted in five out of ten cats with PIB for HAMSP, six out of eight for HAMSA and six out of nine for Control (exclusion of three cats; see Results section). This categorical variable was also used as a factor in Kruskal–Wallis tests for independent samples. Dunn's *post hoc* tests were performed to determine treatment differences. A single-ordered contingency table was used with faecal scores as the ordered columns, while supplement or protein intake levels were the unordered rows. The differences between supplements and the effects of protein intake levels were detected using an exact Kruskal–Wallis test with a χ^2^ test of association.

## Results

Of the study animals, one cat from the HAMSA group was excluded during the study due to medical problems unrelated to the experiment. Data obtained from two other cats (one from the HAMSA group, one from the Control group) were excluded from the statistical analyses due to post-experimental death of the cats unrelated to the experiment. Multiple outliers were noticed in the datasets of the latter cats, which may be explained by the underlying medical problems.

### Nutrient intake, body weight and apparent protein digestibility coefficients

Data are presented in Table 2. Daily energy, crude protein and crude fat intakes were below the offered amounts for most of the cats, but did not differ between supplements (*P* = 0·744, 0·148 and 0·805, respectively). Logically, energy and crude fat intakes were positively correlated with protein intake (*r* 0·900 and 0·800, respectively; *P* < 0·001 for both parameters). All cats, with the exception of two neutered males, lost weight during the experiment (overall mean of body-weight change over 4-week experiment: 0·28 (sem 0·04) kg; range –0·1 to 0·75 kg), but no effect of supplement was seen (*P* = 0·754). On the contrary, this body-weight difference was negatively correlated with protein intake (*r* –0·842; *P* = 0·000). The apparent protein digestibility coefficients were high in all cats, did not differ between supplements (*P* = 0·372) and were not affected by protein intake level (*P* = 0·808).

**Table 2.** Nutrient intake, body weight loss and apparent protein digestibility coefficients from a feline study on the amino acid-sparing potential of propionic acid (Mean values, pooled standard errors, statistical significance and Pearson correlations with protein intake level (PI))

	Supplement				*P*‖		Pearson correlation	
Parameter	HAMSP*	HAMSA†	Control‡	Pooled sem§	Supplement	PI	*r*	*P*‖
Energy intake (kJ/kg^0·67^ per d)	319·2	251·5	270·9	14·7	0·744	< 0·001	0·900	< 0·001
Crude protein intake (g/kg^0·67^ per d)	5·0	3·9	4·4	0·2	0·148	< 0·001	1·000	< 0·001
Crude fat intake (g/kg^0·67^ per d)	3·6	2·8	3·1	0·2	0·805	< 0·001	0·800	< 0·001
Body weight loss (kg/4 weeks)	0·2	0·4	0·3	0·0	0·754	< 0·001	–0·842	< 0·001
Apparent protein digestibility (%)	87·1	91·3	89·3	1·1	0·372	0·808	NP	NP

HAMSP, propionylated high-amylose maize starch; HAMSA, acetylated high-amylose maize starch; NP, analysed non-parametrically, hence no correlation was calculated.

* HAMSP (*n* 10): Propionylated Hylon VII high-amylose maize starch (National Starch & Chemical Company).

† HAMSA (*n* 8): Acetylated Hylon VII high-amylose maize starch (National Starch & Chemical Company).

‡ Control (*n* 9): Celite 545 (VWR International).

§ Standard error of the mean of data grouped over all supplements.

‖ Statistical significance is set at *P* < 0·05.

### Faecal parameters

Faecal parameters are shown in Table 3. Faecal pH differed among supplements (*P* = 0·031), with *post hoc* tests revealing significantly lower values for HAMSP as compared with HAMSA (*P* = 0·043) and independent of protein intake level (*P* = 0·228). Overall, faecal consistency scores indicated rather wet and soft faeces, combined with a low total faecal DM % for all three supplements. For the faecal consistency score, again, no differences between supplements (*P* = 0·122) were observed, even when corrected for protein intake level (*P* = 0·473). However, when faecal consistency scores were grouped into three categories (too hard, normal, too soft; data not shown), HAMSP demonstrated a higher incidence of too-soft faeces as compared with HAMSA (*P* = 0·031), independent of protein intake level (*P* = 0·473). Faecal DM % correlated negatively with protein intake (*r* –0·464; *P* = 0·015), whereas total faecal production over 5 d correlated positively with protein intake (*r* 0·591; *P* = 0·001). Due to the latter positive correlation, the faecal fermentation endproducts were expressed as absolute excretions over 5 d, instead of concentrations in the fresh faecal samples. Faecal acetic, propionic, butyric, valeric, isobutyric and isovaleric acid, and ammonia excretions were positively correlated with protein intake (for *r* and *P* values, see Table 3), whereas no significant differences between supplements were observed (Table 3). For faecal indole, *p*-cresol and phenol excretions, no effects of supplement level were seen even when corrected for protein intake level (for *P* values, see Table 3).

**Table 3.** Faecal parameters of a feline study on the amino acid-sparing potential of propionic acid (Mean values, pooled standard errors, statistical significance and Pearson correlations with protein intake level (PI))

	Supplement				*P*‖		Pearson correlation	
Parameter	HAMSP*	HAMSA†	Control‡	Pooled sem§	Supplement	PI	*r*	*P*‖
Faecal pH	5·05^a^	5·93^b^	5·90^a,b^	0·18	0·031	0·228	NP	NP
Faecal consistency score¶	5·57	4·06	4·50	0·31	0·122	0·473	NP	NP
Faecal DM (%)	26·46	32·25	32·49	1·86	0·659	0·043	–0·464	0·015
Faecal production (g/5 d)	105·70	53·25	102·00	14·15	0·484	0·004	0·591	0·001
Excretions of fermentation endproducts over 5 d								
Acetic acid (mmol/5 d)	6·70	3·02	6·63	1·46	0·384	0·006	0·578	0·002
Propionic acid (mmol/5 d)**	3·18	0·81	1·82	0·36	0·130	0·010	0·601	0·001
Butyric acid (mmol/5 d)	3·53	1·76	2·55	0·45	0·940	0·000	0·708	0·000
Valeric acid (mmol/5 d)	1·24	0·50	0·51	0·18	0·456	0·026	0·513	0·006
Isobutyric acid (μmol/5 d)	48·98	53·18	58·00	6·10	0·230	0·003	0·498	0·008
Isovaleric acid (μmol/5 d)	98·00	92·05	92·93	9·89	0·478	0·001	0·608	0·001
Ammonia (mmol/5 d)	21·94	29·10	24·41	3·72	0·148	0·004	0·449	0·019
Indole (mg/5 d)	0·73	0·58	1·22	0·21	0·478	0·658	NS	NS
* p*-Cresol (mg/5 d)	2·22	2·02	2·39	0·35	0·934	0·564	NS	NS
Phenol (mg/5 d)**	3·34	1·96	1·62	0·50	0·152	0·904	NS	NS
Concentrations of fermentation endproducts in fresh faeces								
Acetic acid (μmol/g)	58·19	47·55	50·99	3·84	0·782	NA	NA	NA
Propionic acid (μmol/g)**	27·99^a^	14·24^b^	14·03^b^	2·10	0·009	NA	NA	NA
Butyric acid (μmol/g)	31·72	25·23	20·14	2·81	0·731	NA	NA	NA
Valeric acid (μmol/g)	8·47^a^	7·99^a,b^	4·67^b^	1·26	0·043	NA	NA	NA
Isobutyric acid (μmol/g)	0·57^a^	1·34^b^	0·90^a,b^	0·13	0·184	NA	NA	NA
Isovaleric acid (μmol/g)	1·08^a^	2·20^b^	1·39^a,b^	0·20	0·182	NA	NA	NA
Ammonia (μmol/g)	223·16^a^	530·81^b^	322·86^a^	42·66	0·021	NA	NA	NA
Indole (μg/g)	8·19	23·70	21·69	6·36	0·359	NA	NA	NA
* p*-Cresol (μg/g)	27·95	59·11	40·99	9·77	0·635	NA	NA	NA
Phenol (μg/g)**	43·07	58·54	35·73	7·98	0·563	NA	NA	NA
Faecal bacterial N (% N excretion)	0·94	0·88	0·75	0·04	0·690	0·176	NS	NS

HAMSP, propionylated high-amylose maize starch; HAMSA, acetylated high-amylose maize starch; NP, analysed non-parametrically, hence no correlation was calculated; NS, *P* value for protein intake > 0·1; NA, comparison not applicable due to significant correlation with faecal production.

^a,b^ Mean values within a row with unlike superscript letters were significantly different (*P* < 0·05).

* HAMSP (*n* 10): Propionylated Hylon VII high-amylose maize starch (National Starch & Chemical Company).

† HAMSA (*n* 8): Acetylated Hylon VII high-amylose maize starch (National Starch & Chemical Company).

‡ Control (*n* 9): Celite 545 (VWR International).

§ Standard error of the mean of data grouped over all supplements.

‖ Statistical significance is set at *P* < 0·05.

¶ Purina Faecal Scoring System^(^^21^^)^: 1 → 7 = hard → soft.

** Data subjected to log transformation to resolve heterogeneity of variance.

As total faecal production did not differ between supplements (*P* = 0·484), concentrations of fermentation endproducts in fresh faecal samples could be compared between supplements. In contrast to absolute excretions, the concentrations of propionic acid (overall *P* = 0·009) were higher in HAMSP as compared with HAMSA (*P* = 0·018) and Control (*P* = 0·003), and valeric acid concentrations were higher in HAMSP compared with Control (*P* = 0·043). Additionally, faecal ammonia concentrations were significantly higher in HAMSA as compared with HAMSP (*P* = 0·007) and Control (*P* = 0·043). No differences between supplements were seen for faecal concentrations of acetic acid, butyric acid, indole, *p*-cresol and phenol (for *P* values, see Table 3). No effects of supplement (*P* = 0·690) or protein intake (*P* = 0·176) were seen on faecal bacterial N excretion when expressed in percentage of the total N excretion or of supplement when expressed in concentrations (data not shown; *P* = 0·214).

### Serum and plasma parameters

Data are shown in Table 4. Among plasma amino acid profiles, no significant differences between supplements were seen even when corrected for protein intake level (for *r* and *P* values, see Table 4). Among plasma acylcarnitine profiles, tendencies for higher propionylcarnitine concentrations (overall *P* = 0·086) were observed in HAMSP as compared with HAMSA (*P* = 0·090) and Control (*P* = 0·037), while trends for a lower methylmalonyl:propionylcarnitine ratio (overall *P* = 0·089) were found for HAMSP as compared with HAMSA (*P* = 0·058) and Control (*P* = 0·054). Additionally, trends for higher tiglyl- + 3-methyl crotonylcarnitine concentrations (overall *P* = 0·067) were seen in the plasma of HAMSP (*P* = 0·028) as compared with Control cats. All above-mentioned observations were independent of protein intake level (for *P* values, see Table 4). A negative correlation was found between plasma concentrations of methylmalonyl-, 3-OH butyrylcarnitine and protein intake, whereas plasma acetylcarnitine tended to be negatively correlated with protein intake level (for *r* and *P* values, see Table 4). For creatine kinase, and 1- and 3-methylhistidine, no differences were noted between supplements even when corrected for protein intake level (for *P* values, see Table 4). For serum creatinine concentrations, no effects of supplements (*P* = 0·963) or protein intake level (*P* = 0·747) were seen. Serum urea concentrations did not differ between supplementation groups (*P* = 0·459), whereas a negative correlation was noted with protein intake (*r* –0·442; *P* = 0·021). Serum total protein did not differ between supplements (*P* = 0·828), but a positive correlation was observed with protein intake (*r* 0·453; *P* = 0·018).

**Table 4.** Plasma and serum parameters of a feline study on the amino acid-sparing potential of propionic acid (Mean values, pooled standard errors, statistical significance and Pearson correlations with protein intake level (PI))

	Supplement				*P*‖		Pearson correlation	
Parameter	HAMSP*	HAMSA†	Control‡	Pooled sem§	Supplement	PI	*r*	*P*‖
Plasma amino acid profile (µmol/l)								
Valine	133·44	133·03	138·93	4·65	0·793	0·260	NS	NS
Leucine	134·16	146·11	139·66	4·64	0·913	0·160	NS	NS
Methionine	42·60	41·42	44·21	1·31	0·725	0·985	NS	NS
Phenylalanine	54·85	52·91	51·96	1·48	0·390	0·083	–0·275	0·165
Tyrosine	37·59	41·79	40·27	1·66	0·937	0·177	NS	NS
Ornithine	24·60	24·05	22·61	0·97	0·683	0·764	NS	NS
Citrulline	33·40	37·14	29·06	1·64	0·164	0·147	NS	NS
Glycine	345·31	355·20	349·28	9·67	0·977	0·149	NS	NS
Alanine	699·37	630·26	652·30	21·45	0·372	0·594	NS	NS
Plasma acylcarnitine profile (µmol/l)								
Free carnitine	27·92	29·44	28·43	2·47	0·984	0·327	NS	NS
Acetylcarnitine	7·96	7·36	7·15	0·58	0·432	0·048	–0·329	0·093
Propionylcarnitine	0·27^a^	0·20^b^	0·17^b^	0·02	0·086	0·268	NS	NS
Butyryl- + isobutyrylcarnitine	0·22	0·24	0·22	0·02	0·955	0·539	NS	NS
Methylmalonylcarnitine	0·06	0·06	0·05	0·00	0·740	0·028	–0·459	0·016
Methylmalonyl:propionylcarnitine ratio¶	0·24^a^	0·40^b^	0·36^b^	0·03	0·089	0·749	NS	NS
3-OH 3-CH_3_ glutarylcarnitine	0·01	0·01	0·01	0·00	0·817	0·306	NP	NP
Isovaleryl- + 2-CH_3_ butyrylcarnitine	0·23	0·19	0·23	0·07	0·479	0·149	NS	NS
3-OH isovaleryl- + 2-CH_3_-3-OH butyrylcarnitine	0·10	0·11	0·08	0·05	0·738	0·103	NP	NP
3-OH butyrylcarnitine	0·06	0·06	0·08	0·02	0·546	0·010	–0·502	0·008
Tiglyl- + 3-CH_3_ crotonylcarnitine	0·05^a^	0·05^a^	0·03^b^	0·00	0·067	0·130	NS	NS
Serum creatine kinase (U/l)¶	314·70	189·75	330·00	47·06	0·666	0·506	NS	NS
Plasma 3-methylhistidine (µmol/l)	27·34	29·85	29·54	1·82	0·947	0·517	NS	NS
Plasma 1-methylhistidine (µmol/l)	25·71	24·76	27·75	1·52	0·743	0·507	NS	NS
Serum creatinine (mg/l)	18·1	18·3	17·9	0·7	0·963	0·747	NS	NS
Serum urea (mg/l)	390·0	452·5	414·4	13·2	0·459	0·085	–0·442	0·021
Serum total protein (g/l)	77·5	76·0	77·2	1·5	0·828	0·020	0·453	0·018

HAMSP, propionylated high-amylose maize starch; HAMSA, acetylated high-amylose maize starch; NS, *P* value for protein intake > 0·1; OH, hydroxyl; CH_3_, methyl; NP, analysed non-parametrically, hence no correlation was calculated.

^a,b^ Mean values within a row with unlike superscript letters were significantly different (*P* < 0·05).

* HAMSP (*n* 10): Propionylated Hylon VII high-amylose maize starch (National Starch & Chemical Company).

† HAMSA (*n* 8): Acetylated Hylon VII high-amylose maize starch (National Starch & Chemical Company).

‡ Control (*n* 9): Celite 545 (VWR International).

§ Standard error of the mean of data grouped over all supplements.

‖ Statistical significance is set at *P* < 0·05.

¶ Data subjected to log transformation to resolve heterogeneity of variance.

## Discussion

The present study is the first to investigate the fermentation metabolite and endproduct profiles of acylated starches in domestic cats. High-amylose maize starches esterified with propionic (HAMSP) or acetic (HAMSA) acid were used in this experiment. The aim of feeding these supplements was to compare the metabolic effects of acetic and propionic acids after de-esterification of the latter molecules from the starch residue in the large intestine and absorption into the blood. In general, acetic acid is known not to be a gluconeogenic substrate^(^^19^^)^, whereas propionic acid is considered to be a gluconeogenic substrate post-absorption^(^^6^^)^. Propionic acid, therefore, has the potential of sparing the dietary and endogenous amino acids from participation in gluconeogenesis^(^^3^^,^^4^^)^. Amino acids are channelled at a high rate to gluconeogenesis in domestic cats^(^^24^^)^.

The first prerequisite to enable the study of the supplements' metabolic effects is the de-esterification of acylated starch within the large intestine into SCFA and resistant starch residues. In the rat, the ester bond is degraded by colonic bacterial enzymes. Consequently, acylated starches supply the large intestine with significant quantities of de-esterified SCFA in addition to those derived from the fermentation of resistant starch residues^(^^9^^)^. The present study provided evidence that the HAMSP ester bond is degraded in the feline large intestine, since faecal propionic acid concentrations were the highest in this group. Faecal pH was the lowest in HAMSP, indicative of an extensive de-esterification of propionylated starch as well. Remarkably, faecal acetic acid concentrations were not significantly higher in the HAMSA cats as compared with the other supplements and the faecal concentrations of branched-chain fatty acids and ammonia were highest in HAMSA-supplemented cats.

A possible explanation for the difference in large-intestinal fermentation pattern between HAMSA (proteolytic) and HAMSP (saccharolytic) might lie in the structural difference between the two supplements. Due to the longer chain length of the esterified SCFA in HAMSP in comparison with HAMSA, the molecular structure of HAMSP is more compact than that of HAMSA^(^^11^^)^. Therefore, the ester bond in HAMSA may be more accessible for bacterial enzymes and HAMSA might have been de-esterified already in the feline small intestine before reaching the large intestine, especially since higher numbers of bacteria have been shown to reside in the feline small intestine as compared with other species^(^^25^^–^^27^^)^. The small-intestinal microbial de-esterification of HAMSA and the potential fermentation of resistant starch residues within the small intestine might have lowered the pH in this region of the gastrointestinal tract. A decreased small-intestinal luminal pH would impair the optimal functioning of endogenous digestive enzymes^(^^28^^)^, impairing small-intestinal protein digestion and stimulating large-intestinal protein fermentation, reflected in the high levels of ammonia, branched-chain fatty acids, phenol and *p*-cresol in the faeces of cats consuming the HAMSA supplement. It has to be noted that the present study is the first at our laboratory that detected phenol in feline samples due to optimisation in terms of sensitivity of the analysis protocol compared with previous studies^(^^4^^,^^5^^,^^7^^,^^8^^)^. The apparent protein digestibility coefficients did not differ between groups, but these parameters reflect total-tract rather than small-intestinal digestibility.

The second prerequisite is the absorption of SCFA from the intestine into the blood, which can be estimated based on plasma acylcarnitine profiles. In the plasma of HAMSP-supplemented cats, concentrations of propionylcarnitine were higher (in comparison with Control) or tended to be higher (in comparison with HAMSA) compared with other supplements, consistent with a (tendency towards a) higher propionic acid absorption from the large intestine. Hence, a higher availability of propionic acid for hepatic metabolism may be assumed. This expected result shows that a sufficient intake level of the dietary supplements had been achieved. Higher propionylcarnitine concentrations were not accompanied by a rise in plasma concentrations of methylmalonylcarnitine in HAMSP-supplemented cats. Methylmalonyl-CoA, measured in plasma as methylmalonylcarnitine, can be produced from fermentation-derived propionic acid or upon degradation of valine and isoleucine^(^^29^^)^. A concomitant increase in propionylcarnitine through fermentation and a lack of increase of methylmalonylcarnitine might thus be due to a sparing of valine and isoleucine^(^^3^^,^^4^^)^. In contrast, the higher plasma tiglyl- + 3-methylcrotonylcarnitine concentrations in HAMSP, as compared with the Control group, are consistent with a higher endogenous leucine and isoleucine breakdown in the former group^(^^29^^)^. The sparing of isoleucine by fermentation-derived propionic acid is, therefore, questionable in this experiment.

It has to be noted that the mean energy intake of both treatment groups and the Control group was considerably lower than the calculated maintenance energy requirements (418·4 kJ/kg^0·67^; see National Research Council^(^^18^^)^). This energy intake was not sufficient to maintain body weight in all cats, and for more than half of the cats (seventeen out of twenty-seven; five in HAMSP, six in HAMSA, six in Control), crude protein intake was below the recommended minimal requirements. As a consequence, mean intakes of most amino acids were below the adequate or minimum intake requirements as well^(^^18^^)^. Since no significant differences in energy, protein, fat or amino acid intakes were observed between groups, the comparison between supplements as described above are valid, but only applicable for situations of relative protein shortage and energy intakes below maintenance energy requirements. The protein intake level was, therefore, included as a covariate in the statistical analyses, and the data from the cats with a low protein intake might serve as a model for diseased cats in clinical circumstances with a low food and protein intake. Especially under these circumstances, the amino acid-sparing potential of propionic acid would be advantageous. Of course, the simple extrapolation of data from healthy cats with a low energy and protein intake to disease-afflicted cats may be confounded by the various metabolic differences existing between healthy and diseased cats. Follow-up studies, quantifying the amino acid-sparing effects of propionate in healthy cats with a range of dietary protein intakes and in cats in various disease states, will be important.

The results from the present study indicate that increased propionic acid from dietary supplementation had no impact on amino acid sparing in cats with a low dietary protein intake, since plasma methylmalonylcarnitine concentrations were negatively correlated with protein intake levels. This is in accordance with higher plasma concentrations of this carnitine when protein intake was low. When protein intake was low, a higher catabolism of valine and isoleucine was expected, overriding the potential sparing of these amino acids by propionic acid. The latter cats showed other signs of a higher endogenous protein catabolism to fulfil the metabolic demand for N and energy precursors as well, such as higher concentrations of serum urea and plasma concentrations of all measured free amino acids above the plasma concentrations of kittens fed diets containing each amino acid at minimal requirement^(^^30^^)^. However, no significant correlations were found between the protein intake and other carnitines that represent the catabolism of branched-chain amino acids^(^^29^^)^: tiglyl- + 3-methylcrotonylcarnitine, isovaleryl- + 2-methylbutyrylcarnitine, 3-OH isovaleryl- + 2-methyl-3-OH butyrylcarnitine from leucine and isoleucine. Likewise, creatinine, creatine kinase and 3-methylhistidine were not affected by protein intake level. However, the latter parameters are not sensitive or affected by other factors, such as stress or restraint of the animal^(^^31^^–^^33^^)^. In contrast, in cats consuming adequate levels of protein, increased dietary propionic acid was associated with signs of sparing of the amino acid valine (see above).

Another remarkable effect of the low protein intake was the higher plasma concentrations of 3-OH butyrylcarnitine combined with a lower faecal butyric acid excretion. This carnitine ester is an estimator of the concentration of 3-OH butyryl-CoA, which is a metabolite in the β-oxidation pathway in colonocytes. In this pathway, acetyl-CoA and ketone bodies are produced from large-intestinal fermentation-derived butyric acid to yield energy for the colonocytes^(^^34^^)^. A possible explanation for the effect of protein intake level on this parameter is that faecal butyric acid excretion and, by extrapolation, large-intestinal butyric acid production were lower when protein intake was lower. Large-intestinal butyric acid is known to stimulate the mRNA expression of an important rate-limiting enzyme in the β-oxidation pathway, namely 3-OH 3-methylglutaryl-CoA (HMG) synthase^(^^35^^,^^36^^)^. A consequence of lower concentrations of butyric acid is a lower activity of HMG synthase and accumulation of metabolites higher in the pathway, including 3-OH butyryl-CoA, which can be absorbed into the blood^(^^37^^)^ and was detected via higher plasma concentrations of 3-OH butyrylcarnitine. The lower activity of HMG synthase can also explain why an increase in 3-OH butyryl-CoA is not accompanied by an increase in HMG. These observations contrast with previously published results^(^^8^^)^, wherein a higher ratio of faecal butyric acid to total SCFA in guar gum-, as compared with cellulose-, supplemented cats was accompanied by higher concentrations of plasma 3-OH butyrylcarnitine. From this discrepancy it can be concluded that 3-OH butyryl-CoA is a metabolite from β-oxidation in colonocytes, which appears to be rapidly absorbed into the hosts' blood in cases of high concentrations in colonocytes by a higher production (as described by Rochus *et al.*^(^^8^^)^) or an accumulation (as in the present study).

### Conclusions

The HAMSP supplement produced a different, more saccharolytic, fermentation pattern in the feline large intestine as compared with HAMSA. The HAMSP-supplemented cats appeared to show sparing of valine, whereas HAMSA- and Control-fed cats did not. The energy and protein intake of all cats was below the offered amounts and in cats with a low protein intake the amino acid-sparing potential of propionic acid was not sufficient to compensate for the higher endogenous protein catabolism. Further studies to explore the ideal dose of dietary HAMSP supplementation and to quantify the amino acid-sparing effect in domestic cats are warranted.
